# Serum prealbumin and its changes over time are associated with mortality in acute kidney injury

**DOI:** 10.1038/srep41493

**Published:** 2017-02-01

**Authors:** Wenji Wang, Yu Pan, Xiao Tang, Guihua Hao, Yingxin Xie, Shuai Ma, Jianfeng Luo, Daqiao Guo, Feng Ding

**Affiliations:** 1 Division of Nephrology, Shanghai Ninth People’s Hospital, School of Medicine, Shanghai Jiaotong University, Shanghai 200011, China; 2 Institute of Vascular surgery, Division of Vascular surgery, Zhongshan Hospital, Fudan University, Shanghai 200032, China; 3 Department of Biostatistics, Public Health School, Fudan University, Shanghai 200032, China.

## Abstract

Serum prealbumin is a clinically relevant indicator of nutritional status and inflammation in patients with acute kidney injury (AKI). This study aimed to determine whether serum prealbumin and its longitudinal changes over a week could improve the prediction of 90-day mortality in AKI patients. This prospective cohort study included 340 adults with AKI between 2014 and 2015. There were 94 (27.6%) patient deaths within 90 days. Serum prealbumin level <10 mg/dL at the time of AKI diagnosis was associated with a 155% increased death risk ratio (adjusted hazard ratio [HR], 2.55; 95% confidence interval [CI], 1.18 to 5.49; *P* = 0.02). Serum prealbumin fall >4 mg/dL was also associated with 90-day mortality in adjusted Cox regression models (HR, 1.79; 95% CI, 1.06 to 3.03; *P* = 0.03). Compared to serum albumin, mortality-predictability of serum prealbumin (*P* = 0.01) and its changes (*P* = 0.01) were both increased. Adding prealbumin and its changes on the conventional covariates improved the prediction of progression to 90-day mortality (NRI 0.29, *P* = 0.04; aIDI 0.08; *P* = 0.03). In conclusion, serum prealbumin, and its changes were independent predictors of worse prognosis in AKI, and could be potential surrogates to better predict 90-day mortality.

Acute kidney injury (AKI) is a well-known problem in hospitalized patients, especially in serious patients, and is associated with poor outcomes^1^,^2^. Despite advances in the diagnosis and treatment of AKI, mortality from this condition remains high^3^,^4^. Inflammation and protein-energy wasting (PEW) are common among patients with AKI and are strongly associated with all-cause mortality^5^,^6^,^7^.

According to the International Society of Renal Nutrition and Metabolism, the serum biochemical markers including albumin, prealbumin, and cholesterol are recommended as markers for assessing the nutritional status^8^,^9^. The established evidence indicated that hypoalbuminemia is a significant independent predictor for both AKI and the related mortality^8^. Recently, several studies have advocated that serum prealbumin, also known as transthyretin, is a better marker for nutritional status evaluation and an indicator of inflammation in patients with acute and chronic kidney disease, as well as haemodialysis^9^,^10^,^11^. Compared to albumin, prealbumin has a shorter half-life, a more rapid rate of hepatic synthesis, and predictable catabolic rate^12^; hence, it may be a more sensitive indicator^13^. However, whether prealbumin could be a prognostic indicator is still unclear and the longitudinal changes of serum prealbumin over time have not been studied. We previously reported that the ratio of high-sensitivity C-reactive protein (hsCRP) to prealbumin concentration could predict the 28 days mortality in patients with AKI (n = 150)^8^. In the current study, we evaluated whether serum prealbumin could predict the 90-day mortality in hospital-acquired AKI patients after controlling for other nutritional and inflammatory markers.

In this context, we hypothesized that variation in serum prealbumin concentrations over time are related to the 90-day mortality in AKI, and that adds to the predictive benefit of prealbumin and its changes compared to the conventional predictors, such as serum albumin, cholesterol, serum creatinine, and hsCRP.

## Results

### Clinical and biochemical characteristics of the study subjects

**Figure 1:**
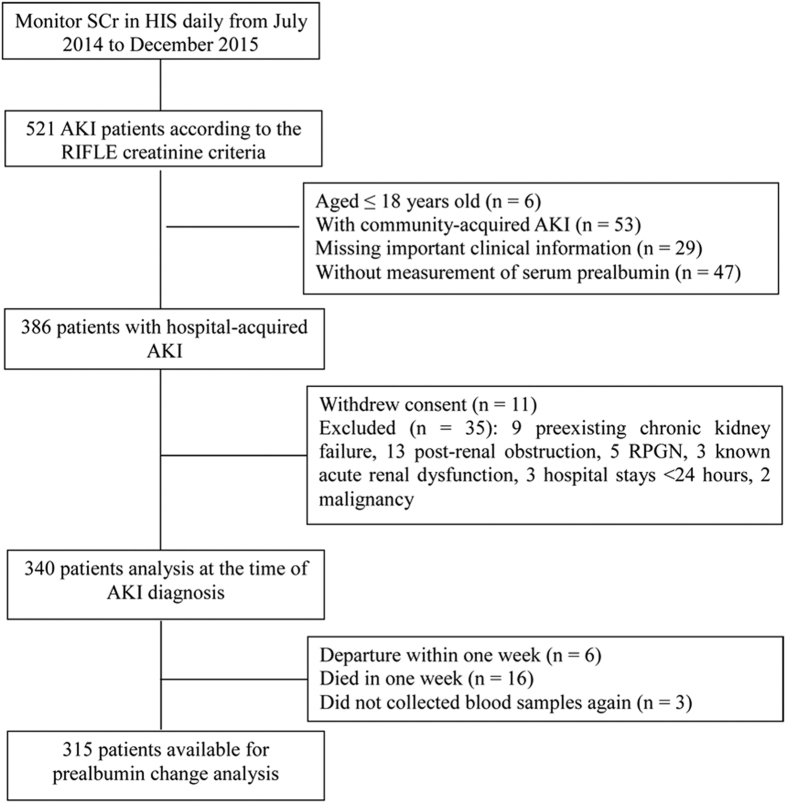
Flow chart of study progress. SCr, serum creatinine; HIS, Hospital Information System; AKI, acute kidney injury; RPGN, rapidly progressing glomerulonephritis.

After preliminary screening, 386 patients enrolled. Due to the exclusion criteria, 35 patients were rejected. Meanwhile, 11 patients with AKI withdrew consents. Then, 340 patients with AKI remained. During information analysis and follow-up, 25 patients left this study, including 6 cases of departure within 1 week, 3 cases of blood sample deficiency, and 16 cases of death in 1 week. As a result, 315 patients with AKI qualified for the prealbumin change analysis, and the flow chart of enrolment is shown in Fig. 1. The average serum prealbumin of patients was 17.64 ± 6.88 mg/dL (median, 17.00; interquartile range, 11.05 to 22.85 mg/dL) at AKI diagnosis. *A priori* categories of serum prealbumin concentrations with 5 mg/dL increments were selected: <10 mg/dL (n = 67; 19.7%), 10 to <15 mg/dL (n = 70; 20.6%), 15 to <20 mg/dL (n = 79; 23.2%), and ≥20 mg/dL (n = 124; 36.5%). Patients’ demographics, clinical features, and laboratory measures per the four categories of prealbumin are shown in Table 1, along with the in-hospital mortality and 90-day mortality. Among these four groups, patients with higher prealbumin levels presented a high ratio of operation but lower ratios of in-hospital mortality, 90-day mortality, and respiratory failure (p < 0.05). Meanwhile, the ratios of disseminated intravascular coagulation and coma were also lower inpatients with high prealbumin levels (p < 0.05) among these four groups. Moreover, patients with lower prealbumin levels had more comorbidities according to the APACHE II score. However, there were no significant results identified in heart failure, hepatic failure, infection, hypotension, haemorrhage of digestive tract, cause of AKI, and platelet count among these four groups (p < 0.05).

### Alteration of prealbumin over 1 week was associated with 90-day mortality

**Figure 2:**
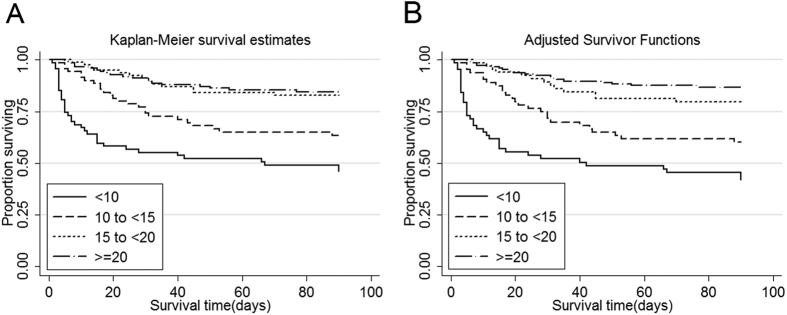
Kaplan-Meier proportion of surviving patients after 90 days of observation according to the four *a priori*-selected groups of serum prealbumin in 340 patients with AKI. (**A**) Unadjusted; (**B**) adjusted for age, sex, hypertension, hypertension, APACHE II score, continuous renal replacement therapy, serum albumin, white blood cell count, and serum high-sensitivity C-reactive protein. Serum prealbumin cut-offs are <10 mg/dL, 10 to <15 mg/dL, 15 to <20 mg/dL, and ≥20 mg/dL. AKI, acute kidney injury; APACHE, Acute Physiology, and Chronic Health Evaluation.

The total in-hospital and 90-day mortality rates were 20.3% and 27.6% in all 340 patients, respectively. Correlations between serum prealbumin and other relevant variables conducted by the Pearson method are shown in Table 2. Among the clinical variables, a significant correlation was identified in serum prealbumin with APACHE II score (p < 0.001), but no remarkable correlations were identified in serum prealbumin with age and RIFLE (Risk, Injury, Failure, Loss, and End-stage) (p > 0.05). Meanwhile, among the biochemical variables obvious correlations were also observed between serum prealbumin and albumin, cholesterol, calcium, haemoglobin, and hsCRP (p < 0.01), but not phosphorus (p > 0.05). In order to reveal the correlation between serum prealbumin and mortality, the Kaplan-Meier analysis was performed. As a result, significant elevated mortality were identified in both in-hospital and 90-days groups with serum prealbumin levels <10 mg/dL and 10 to <15 mg/dL (p < 0.001) (Fig. 2A). In addition, these significant elevations were identified in adjusted in-hospital and 90-days groups with serum prealbumin levels <10 mg/dL and 10 to <15 mg/dL (p < 0.001) (Fig. 2B). Moreover, the Cox regression analysis of serum prealbumin and adjusted multivariate was also carried out. The analysed results showed that a serum prealbumin level of <10 mg/dL was associated with a 155% increased death risk ratio (hazard ration (HR), 2.55; 95% CI, 1.18 to 5.49; p = 0.02) (Table 3).

**Figure 3:**
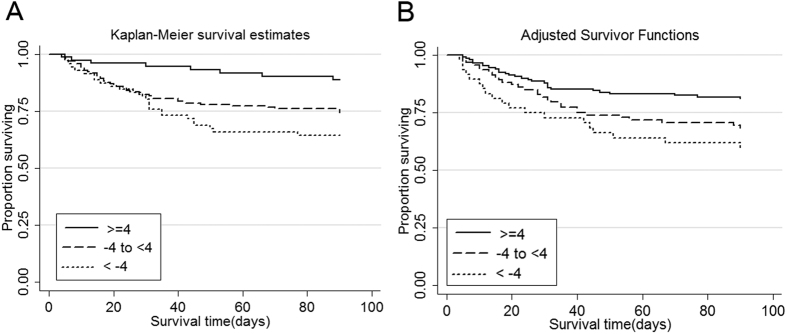
Kaplan-Meier proportion of surviving patients after 90 days of observation according to the three *a priori*-selected groups of changes in serum prealbumin in 315 patients with AKI. (**A**) Unadjusted; (**B**) adjusted for age, sex, hypertension, APACHE II score, hypertension, continuous renal replacement therapy, serum albumin, white blood cell count, and serum high-sensitivity C-reactive protein. Serum prealbumin cut-offs are <−4 mg/dL, −4 to <4 mg/dL, and ≥4 mg/dL. AKI, acute kidney injury; APACHE, Acute Physiology, and Chronic Health Evaluation.

**Figure 4:**
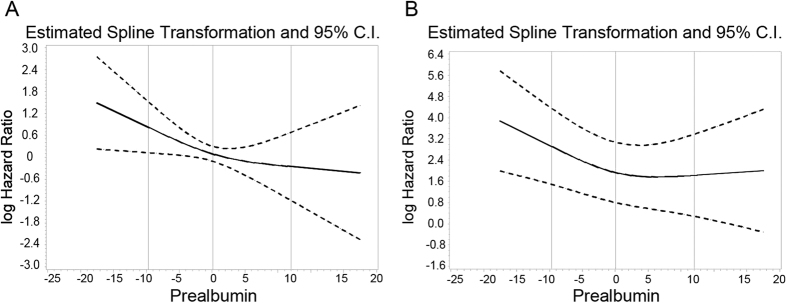
Mortality predictability of changes in serum prealbumin during a 90-day period in 315 patients with AKI. (**A**) Unadjusted; (**B**) fully adjusted for age, sex, hypertension, APACHE II score, continuous renal replacement therapy, serum albumin, white blood cell count, and serum high-sensitivity C-reactive protein. AKI, acute kidney injury; APACHE, Acute Physiology, and Chronic Health Evaluation.

To further examine whether longitudinal changes in serum prealbumin over 1 week could predict in-hospital mortality and 90-day mortality in patients with AKI (n = 315), the magnitude and direction of the change in serum prealbumin levels were calculated. The incidence of in-hospital mortality was significantly higher in patients with a serum prealbumin fall >4 mg/dL compared with those with serum prealbumin rise >4 mg/dL (22.2% versus 7.9%, p = 0.018). A rapid decline in serum prealbumin level also showed a tendency towards increased 90-day mortality (36.1% versus 13.2%, p = 0.005). There were significant differences in 90-day survival among groups of changes in serum prealbumin levels according to the Kaplan-Meier survival curves (Fig. 3). According to the cubic splines, graphs for the associations between changes in serum prealbumin and all-cause 90-day mortality, a rapid decline in serum prealbumin level was associated with increased death risk in the full-adjusted models (Fig. 4). Meanwhile, the HRs of the *a priori*-selected groups for prealbumin change were also conducted and the results are shown in Table 4. Compared to patients with stable prealbumin levels, the patients with serum prealbumin fall >4 mg/dL had a 79% increased death risk ratio (HR 1.79; 95% CI, 1.09 to 3.03; p = 0.03). In addition, among patients with serum prealbumin ≥15 mg/dL when AKI was diagnosed, a serum prealbumin fall >4 mg/dL also showed more than 3-fold increase in all-cause death risk (HR, 3.85; 95% CI, 1.55 to 9.52; p = 0.04).

### Added predictive benefit of prealbumin and its changes over time in the prediction of 90-day mortality

**Figure 5:**
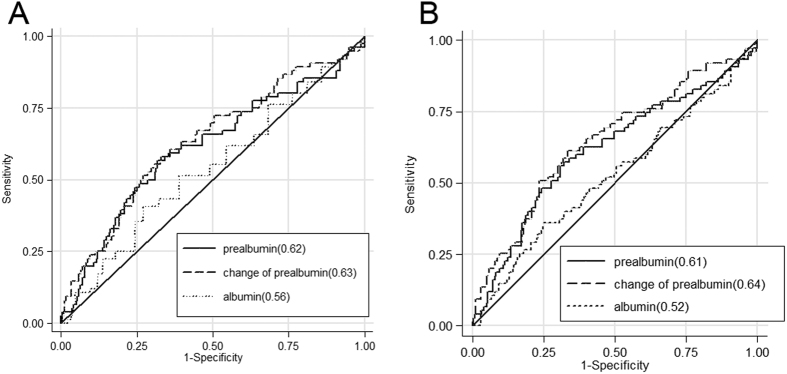
Receiver operating characteristic (ROC) curves of probabilities obtained from hazard regression models of serum albumin, prealbumin, and change in serum prealbumin as independent variables and all-cause mortality as dependent (reference) variable in unadjusted and adjusted formats. A larger area under the curve (AUC) indicates higher prognostic value. (**A**) Unadjusted; (**B**) adjusted for age, sex, hypertension, APACHE II score, hypertension, continuous renal replacement therapy, serum albumin, white blood cell count, and serum high-sensitivity C-reactive protein. APACHE, Acute Physiology, and Chronic Health Evaluation.

To compare the mortality predictability of serum albumin, prealbumin, and prealbumin change with each other, receiver-operating characteristics (ROC) curve analyses were performed. As shown in Fig. 5, the mortality-predictability of serum prealbumin seemed to be similar with that of albumin in unadjusted models (AUCs, 0.62 versus 0.56; p = 0.37), but prealbumin changes had a better mortality-predictability than albumin (AUCs, 0.63 versus 0.56; p = 0.01) (Fig. 5A). However, using fully adjusted multivariables, mortality-predictability of both serum prealbumin and its changes were higher than albumin (AUCs, 0.61 versus 0.52; p = 0.01; AUCs, 0.64 versus 0.52; p = 0.01, respectively) (Fig. 5B). In addition, Cox regressions of conventional covariates, prealbumin, and prealbumin with its changes were performed, and the Harrell c index, net reclassification improvement (NRI), and integrated discrimination improvement (IDI) values for predicting mortality at 90-day are summarized in Table 5. It was noted that there was no significant improvement identified in the prediction of 90-day mortality in prealbumin combined with conventional covariates, including serum creatinine, albumin, cholesterol, and hsCRP, while compared with conventional covariates (NRI 0.17, *P* = 0.19; aIDI 0.03; p = 0.10), but an obviously increased predictability was uncovered in conventional covariates combined with prealbumin withits changes while compared with conventional covariates (NRI 0.29, p = 0.04; aIDI 0.08, p = 0.03).

## Discussion

The current prospective cohort study found that the level of serum prealbumin during AKI diagnosis was correlated with the prognosis (up to 90 days) in patients with AKI. The lower level of serum prealbumin was associated with poor survival. Patients with the highest serum level of prealbumin (≥20 mg/dL) had higher serum albumin and cholesterol level, and lower serum hsCRP level. Of note, the reduction in serum prealbumin level beyond 4 mg/dL over 1 week appeared to be associated with an elevated death risk in 90 days after AKI. Furthermore, a consistent trend of poor survival was observed with the decline in serum prealbumin among patients with relatively higher prealbumin (≥15 mg/dL) at the time of AKI diagnosis. Compared to serum albumin, with fully adjusted multivariable, the mortality-predictability of serum prealbumin and its changes were both increased. Combining prealbumin and its changes withconventional covariates, such as serum creatinine, albumin, cholesterol, and hsCRP, also could improve the accurarcy of 90-day mortality prediction.

This study is one of the few to show a nutritional and inflammatory indicator with outcomes long after the time of hospitalization. It is recognized that patients with AKI have a high risk of PEW, known previously as malnutrition, because of malnutrition and inflammation^6^,^14^,^15^,^16^. Because both an early study^17^ and a recent meta-analysis^18^ suggested that an energy provision of more than 40 kcal/kg/day did not improve nitrogen balance and may increase the risk of nutrition-related side-effects. Hence the patients enrolled in the study were provided food by the nutrition canteen as per the planned nutrition schedule. This ensured a protein intake of 0.8–1.0 g/kg/day and energy provision of about 35 kcal/kg/day. As a result, this study confirmed that PEW was a significant problem in patients with AKI; approximately 76.2% (259/340) patients in this cohort presented with PEW according to the criteria of serum albumin values <3.8 g/dL. This result was similar to those reported previously. Fiaccadori *et al*. identified PEW in 58% of 309 patients with AKI using the subjective global assessment (SGA)^6^, and 60% of the patients with AKI exhibited moderate or severe PEW in Berbel’s study, based on the International Society of Nutrition and renal Metabolism^14^.

Superior to serum albumin, the role of serum prealbumin level as a marker of PEW status even as a negative acute phase reactant is well established^19^,^20^. Because of the short half-life of prealbumin^20^, it may be a more sensitive indicator than serum albumin in chronic kidney disease (CKD) or critically ill patients^21^,^22^. In this study, serum prealbumin and its changes were observed to be superior to serum albumin in predicting the mortality in AKI after fully adjusted multivariable. Some recent studies have shown that the decline in prealbumin is also associated with severity of the underlying disease and with poor prognosis in various chronic or acute diseases^23^,^24^, especially in AKI and critical illness^8^,^14^,^25^. Furthermore, systemic sustained improvement in prealbumin levels was associated with long-term survival in patients undergoing haemodialysis^26^,^27^. Meanwhile, it is also indicated that controlling of specific amino acids in the early course of AKI will draw a certain degree of anti-effect on the development of inflammatory and apoptotic effects in the renal tubular epithelium in animal models with nephrotoxicity and sepsis^28^,^29^, which may reverse AKI and reduce the mortality^5^. Valdevieso *et al*. examined the association between serum prealbumin level and mortality in patients with AKI. It was shown that a serum prealbumin level below the group median (11 mg/dL) was strongly associated with a higher risk of in-hospital mortality, and this mortality decreased by 29% if the level of serum prealbumin increased by 5 mg/dL^25^. However, none of the investigations has reported on the relationship between prealbumin with long-term mortality of AKI. According to the Nutritional Care Consensus Group, serum prealbumin values >15 mg/dL indicates a lower risk for malnutrition^30^. In this study, 137 patients with serum prealbumin values <15 mg/dL had higher in-hospital and 90-day mortalities than those with serum prealbumin values ≥15 mg/dL, and 67 patients with serum prealbumin values <10 mg/dL had higher risk of 90-day mortality.

To our knowledge, there are few studies evaluating the correlation between the changes in nutritional markers or inflammatory markers over time with adverse outcomes in patients with renal diseases or critical illness^13^,^14^,^31^. One study showed that non-survival patients showed a reduction of several markers of nutritional status, such as serum albumin (from 2.3 to 2.0 mg/dL), serum cholesterol (from 122 to 108 mg/dL), and serum transferring (from 1.19 to 1.02 g/dL), from the first day of nephrology evaluation until nephrology discharge^14^. However, they did not evaluate the association between the decline of these markers and mortality. On the contrary, a small sample study (n = 44) reported that serum prealbumin was not a sensitive predictor of nutrition and prognosis in critically ill patients^31^. Another study conducted by Rambod M *et al*. suggested that the change in serum prealbumin over time was a robust death predictor in patients receiving haemodialysis^13^. Their findings reveal that patients with a prealbumin fall >10 mg/dL over 6 months have a 78% increased 5-year death risk ratio compared with patients with stable prealbumin values. In this context, the declining serum prealbumin levels over time after AKI may imply a more severe status and could be associated with adverse outcomes. Our results indicated that a drop in the serum prealbumin level beyond 4 mg/dL over 1 week appeared to increase the 90-day mortality with a 79% death risk ratio. Although the change in SOFA (Sequential Organ Failure Assessment) score was found to have positive relationship with in-hospital mortality in patients with sepsis^32^ whether it could be utilized in AKI still not clear. At least, this study provides us the idea that the change of marker may be superior to baseline marker (or the time of diagnosis) in prediction value in AKI patients.

This study had certain limitations. All samples used in this study were from a single centre. As such, selection bias during enrolment was inevitable. Most patients in this study were men, but the percentages of men among groups were similar (p = 0.831). The cause of death was not recorded, which makes it difficult to establish a link between serum prealbumin and the cause of death. In addition, urine output was an important criterion, which was not available in this study. Thus, the incidence of AKI may be underestimated and the estimation of RIFLE stages may be affected. Here, comparisons between serum prealbumin and other indicator changes over time to predict the 90-day-survival were not conducted. After adjusting for confounding factors, the predictive powers of albumin, prealbumin, and its changes were indeed lower than the general satisfactory threshold, and the adjusted AUC were 0.52, 0.61, and 0.64, respectively. After screening for possible influencing factors, we expected that this result might be generated without considering the follow-up time while ROC curve was depicted. Considering this, the fully adjusted Cox regression and further C-index and NRI analyses were carried out. The analysed result suggested that prealbumin and its change over time were independent risk factors of 90-day-death, while combining prealbumin with conventional covariates (serum creatinine, albumin, cholesterol, and high-sensitivity C-reactive protein) may have a superior predictability than prealbumin or its change alone as well as any other conventional covariates. Of course, this result was only uncovered in a cohort with small sample size and a larger clinical sample is required to further confirm this result. Despite these limitations, our results would provide useful insights into the performance of serum prealbumin and its changes in AKI.

In conclusion, this study confirmed the relationship between serum prealbumin levels and several markers of PEW and inflammation in patients with AKI and showed that a lower level of serum prealbumin was independently associated with increased death risk. More importantly, data from this study indicated that a decline in serum prealbumin over 1 week was also an independent predictor of worse prognosis. Serum prealbumin and its changes could be potential surrogates to better predict the 90-day mortality in patients with AKI. It was unknown whether this adverse prognostic association was primarily caused by malnutrition, a negative acute phase response to inflammation, transcapillary leak, or a combination of factors. These results may lead to more useful strategies to identify patients at high risk of all-cause death and to develop interventions focusing on the improvement of nutritional or inflammatory status. Further controlled studies are required to examine the exact correlation between serum prealbumin and the severity of diseases or outcomes in patients with AKI.

## Methods

### Patient population and definitions

From July 2014 to December 2015, consecutive cases of adult patients aged >18 years, with hospital-acquired AKI were prospectively entered into a computerized cohort study. The serum prealbumin levels of all patients were measured when AKI was diagnosed and other clinical information was collected. Patients were excluded if they met the following criteria: ① with pre-existing chronic kidney failure (defined as an estimated glomerular filtration rate (eGFR) <60 mL/min per 1.73 m^2^), ② with post-renal obstruction or rapid progressive glomerulonephritis as the main cause of AKI, ③ with known acute renal dysfunction, ④ with hospital stays <24 h ⑤ malignancy. AKI was determined using the RIFLE (Risk, Injury, Failure, Loss and End-stage) creatinine criteria^33^ (at least a 50% increment with respect to the lowest serum level before AKI diagnosis in the hospital). Baseline estimated GFR was calculated by applying the Modification of Diet in Renal Disease (MDRD) equation, with adjustment for female patients^34^. This study was approved by the hospital ethics committee of Shanghai Ninth People’s Hospital, School of Medicine, Shanghai Jiaotong University (approval number: [2014]45). All experimental protocols were performed in accordance with the relevant guidelines and regulations. Written informed consent was obtained from either patients’ legal guardians or themselves prior to participation, in accordance with the Declaration of Helsinki.

### Data collection and study protocol

This is a prospective cohort study. In-patients’ serum creatinine data were monitored in the hospital’s information system (HIS) daily. Patients with creatinine levels rising within 1 week in accordance with RIFLE criteria were consulted by nephrologists within 24 h. Data were collected daily by nephrologists and all collected data were extracted from the electronic medical records. For each patient, the investigators entered the data into a computer using EpiData software. The following information was recorded: demographic characteristics (age, sex, comorbid conditions, cause of AKI, medical, surgery), origin (ward, ID number), severity of illness (evaluated on the first day of AKI diagnosis using Acute Physiology and Chronic Health Evaluation [APACHE] II score)^35^, and renal measures (serum creatinine, RIFLE stage, renal replacement therapy). Comorbidities included heart failure, hypotension, respiratory failure (need for mechanical ventilatory support), acute liver failure (alerted sensorium with the prothrombin time prolonged >4 seconds or INR ≥1.5 or total bilirubin >171 μmol/L)^36^, sepsis (≥2 criteria for Systemic Inflammatory Response Syndrome and proven or suspected infection)^37^, hemorrhage of digestive tract, disseminated intravascular coagulation, and coma (Glasgow Coma Score <8/15)^38^. Biochemical measurements (at the time of AKI diagnosis) were also recorded, including serum albumin, prealbumin, cholesterol, calcium, phosphorus, total bilirubin, high-sensitivity C-reactive protein (hsCRP), haemoglobin, white blood cell count, and platelet count. Blood samples were collected after 7 days to obtain the concentration of serum prealbumin again. All laboratory measurements were performed in the hospital’s Clinical Laboratory with the use of automated methods.

Based on the exclusion criteria, patients were enrolled while AKI was confirmed. All patients enrolled in the study were provided food by the nutrition canteen as per the planned nutrition schedule. This ensured a protein intake of 0.8–1.0 g/kg/day and energy provision of about 35 kcal/kg/day. Consequently, the prospective follow-up was carried out from the time of nephrologists’ consultation until death. The observational period was 90 days and the primary outcome was all-cause mortality. We then restricted our analysis to patients with serum samples at the seventh day after the AKI diagnosis, in order to examine the implication of longitudinal changes in serum prealbumin over time.

### Statistical Analyses

This analytical plan followed the STROBE recommendations for observational cohort studies^39^. The study design mandated accrual of 312 patients, based on an assumed 90-day mortality of 15.0% for patients with higher level of serum prealbumin when AKI was diagnosed, to detect a 20.0% difference in all-cause 90-day mortality (HR of 2.0, 80% power at the 5.0% level of significance)^25^,^40^.

Categorical variables were summarized as frequencies (percentage). Continuous variables were expressed as mean ± standard difference (SD) or medians with interquartile ranges. Comparisons of patients in the *a priori*-selected groups relied on the Chi-squared tests for categorical data and on the ANOVA test or Kruskal-Wallis test, as appropriate, for continuous variables.

Kaplan-Meier analyses were used to assess the differences in the surviving proportions between categories of prealbumin or its changes. To calculate the relative risks of all-cause death, HRs were obtained using the Cox proportional hazard models after controlling for confounding variables. Univariate Cox regression was performed to find potential confounding variables including baseline comorbidities (diabetes mellitus, coronary heart disease, and hypertension), sepsis, continuous renal replacement therapy, APACHE II score, and biochemical measures (serum albumin, total cholesterol, calcium, phosphorus, hsCRP, haemoglobin, and white blood cell count). The multivariable Cox regression model consisted of age, sex, hypertension, APACHE II score, continuous renal replacement therapy, serum albumin, white blood cell count, and serum hsCRP, according to p-value in the univariable regression. Nonlinear associations of continuous mortality predictors were studied using restricted cubic splines to examine the inappropriate linearity assumptions.

Receiver-operating characteristics (ROC) curves and the areas under the curves (AUCs) were used to compare the mortality predictability of serum albumin, prealbumin, and its change. The all-cause death was the reference variable and the unadjusted or fully adjusted death hazard score of serum albumin, prealbumin, or its change was the predicting variable.

To investigate the incremental predictive power of prealbumin and its change to conventional progression models including serum albumin, cholesterol, serum creatinine, and hsCRP, we compared the Harrell concordance index (c index) between multivariate Cox proportional hazards models adjusted for the covariates age, sex, hypertension, APACHE II score, continuous renal replacement therapy, and white blood cell count. Improvement in discriminating the risk of the outcome at 90 days was assessed by the analysis of the category-free NRI and IDI obtained by 10-fold cross-validating using 10,000 bootstrap repetitions of the whole data set^41^,^42^.

All p values are two tailed, and p values <0.05 are considered significant. Statistics were performed with SAS version 9.0 (SAS Inc., Cary, NC) and package R version 2.15.0 (www.r-project.org).

## Additional Information

**How to cite this article**: Wang, W. *et al*. Serum prealbumin and its changes over time are associated with mortality in acute kidney injury. *Sci. Rep.* **7**, 41493; doi: 10.1038/srep41493 (2017).

**Publisher's note:** Springer Nature remains neutral with regard to jurisdictional claims in published maps and institutional affiliations.

## Figures and Tables

**Table 1:** Demographic, clinical, laboratory values, and mortality for total serum prealbumin level and according to the 4 *a priori*-selected groups of serum prealbumin levels in 340 patients at the time of AKI diagnosis.

	Total	<10 mg/dL	10 to <15 mg/dL	15 to<20 mg/dL	≥20 mg/dL	*P*
	n = 340	n = 67	n = 70	n = 79	n = 124	*value*
In-hospital mortality, n %	69 (20.3)	31 (46.3)	16 (22.9)	9 (11.4)	13 (10.5)	<0.001
90 d mortality, n %	94 (27.6)	36 (53.7)	25 (35.7)	13 (16.5)	20 (16.1)	<0.001
Demographic						
Age, y	62.5 ± 16.5	65.0 ± 17.4	66.0 ± 13.2	60.9 ± 16.6	60.2 ± 17.2	0.46
Male, n %	247 (72.6)	50 (74.6)	53 (75.7)	55 (69.6)	89 (71.8)	0.831
Diabetes mellitus, n %	27 (7.9)	5 (7.5)	6 (8.6)	7 (8.9)	9 (7.3)	0.972
Hypertension, n %	94 (27.6)	6 (9.0)	15 (21.4)	30 (38.0)	43 (34.7)	<0.001
CHD, n %	51 (15.0)	3 (4.5)	8 (11.4)	20 (25.3)	20 (16.1)	0.003
Operation, n %	210 (61.8)	29 (43.3)	42 (60.0)	57 (72.2)	82 (66.1)	0.002
Comobidities						
Heart failure, n %	61 (17.9)	7 (10.4)	9 (12.9)	18 (22.8)	27 (21.8)	0.099
Respiratory failure, n %	39 (11.5)	13 (19.4)	7 (10.0)	12 (15.2)	7 (5.6)	0.023
Acute liver failure, n %	9 (2.6)	4 (6.0)	1 (1.4)	2 (2.5)	2 (1.6)	0.285
Sepsis, n %	58 (17.1)	15 (22.4)	13 (18.6)	11 (13.9)	19 (15.3)	0.518
Hypotension, n %	48 (14.1)	12 (17.9)	7 (10.0)	12 (15.2)	17 (13.7)	0.432
Hemorrhage of digestive tract, n %	16 (4.7)	4 (6.0)	4 (5.7)	3 (3.8)	5 (4.0)	0.695
DIC, n %	8 (2.4)	5 (7.5)	1 (1.4)	1 (1.3)	1 (0.8)	0.023
Coma, n %	40 (11.8)	14 (20.9)	9 (12.9)	9 (11.4)	8 (6.5)	0.031
RIFLE criteria, n %						<0.001
Risk	204 (60.0)	27 (40.3)	39 (55.7)	54 (68.4)	84 (67.7)	
Injury	77 (22.6)	17 (25.4)	14 (20.0)	19 (24.0)	27 (21.8)	
Failure	59 (17.4)	23 (34.3)	17 (24.3)	6 (7.6)	13 (10.5)	0.023
CRRT, n %	47 (13.8)	16 (23.9)	10 (14.3)	9 (11.4)	12 (9.7)	0.049
APACHE II score	14 (10, 21)	21 (15, 29)	16 (11, 22)	12 (9, 16)	12 (9, 16.5)	<0.001
≥15, n %	160 (47.1)	51 (76.1)	38 (54.3)	31 (39.2)	40 (32.3)	
<15, n %	180 (52.9)	16 (23.9)	32 (45.7)	48 (60.8)	84 (67.7)	
Cause of AKI, n %						
Prerenal	135 (39.7)	29 (43.3)	23 (32.9)	39 (49.4)	44 (35.5)	0.125
Renal	93 (27.4)	21 (31.3)	20 (28.6)	19 (24.1)	33 (26.6)	0.965
Prerenal + renal	112 (32.9)	17 (25.4)	27 (38.6)	21 (26.6)	47 (37.9)	0.135
Biochemical measurements						
Serum						
Albumin, g/L	33 (29, 37)	29 (24, 33)	32 (29, 35)	34 (29, 37)	36 (33, 39)	<0.001
Creatinine, μmol/L	158 (127, 213)	176 (148, 290)	159 (137, 235)	146 (122, 183)	153 (121, 185)	<0.001
Cholesterol, mmol/L	3.74 ± 1.37	2.85 ± 1.39	3.52 ± 1.13	3.00 ± 1.13	4.27 ± 1.34	<0.001
Calcium, mmol/L	2.08 ± 0.24	2.02 ± 0.30	2.03 ± 0.24	2.04 ± 0.19	2.16 ± 0.20	<0.001
Phosphorus, mmol/L	1.34 ± 0.51	1.27 ± 0.54	1.26 ± 0.54	1.23 ± 0.49	1.43 ± 0.46	0.003
ALT, IU/L	26 (18, 57)	32 (18, 79)	27 (18.5, 62.5)	27 (18, 52)	24 (17, 45)	0.108
Total bilirubin, μmol/L	13.9 (10.0, 23.1)	18.5 (11.0, 40.7)	13.7 (9.7, 21.3)	13.9 (10.6, 24.5)	13.1 (9.0, 18.8)	0.098
hsCRP, mg/L	70.2 (24.9, 127.7)	118.0 (41.2, 193.0)	94.1 (25.8, 128.3)	83.6 (51.7, 135.0)	37.2 (8.9, 78.0)	0.002
Blood						
Hemoglobin, g/L	108.5 ± 21.2	100.0 ± 22.6	110.4 ± 20.3	106.6 ± 18.7	112.8 ± 21.4	0.001
WBC count, × 10^9^ cell/L	13.51 ± 6.64	14.05 ± 5.69	13.47 ± 9.69	13.25 ± 6.10	12.95 ± 5.21	0.686
Lymphocyte, % of WBC	7.8 (5.0, 11.7)	8.0 (5.0, 12.0)	7.1 (4.7, 10.6)	7.5 (4.8, 11.1)	8.3 (5.6, 12.0)	0.334
Platelet count, × 10^12^/L	153.7 ± 90.4	147.5 ± 109.3	155.3 ± 96.7	139.2 ± 75.1	165.4 ± 83.7	0.220

AKI, acute kidney injury; CHD, coronary heart disease; CRRT, continuous renal replacement therapy; ALT, alanine aminotransferase; hsCRP, high sensitivity C-reactive protein; WBC, white blood cell. DIC, disseminated intravascular coagulation.

Continuous variables are expressed as mean ± SD or median (percentiles 25 to 75); categorical variables are expressed as total number and percentage of the global population.

ANOVA or Kruskal-Wallis test and Chi-square test for trend were used for examining the significance of trends in continuous and categorical variables, respectively.

**Table 2:** Unadjusted and multivariate adjusted Pearson’s correlation coefficient of serum prealbumin and other relevant variables in 340 patients with AKI.

	Unadjusted (*P*)	Age and gender adjusted (*P*)	Full model adjusted (*P*)
Clinical variables			
Age	−0.11 (0.016)	0.08 (0.651)	0.07 (0.291)
RIFLE stage	−0.21 (<0.001)	−0.20 (0.703)	−0.18 (0.024)
APACHE II score	−0.26 (<0.001)	−0.26 (<0.001)	−0.07 (0.418)
Biochemical variables			
Albumin	0.12 (0.011)	0.39 (<0.001)	0.29 (<0.001)
Creatinine	−0.17 (0.001)	−0.18 (0.001)	−0.14 (0.071)
Cholesterol	0.43 (<0.001)	0.41 (<0.001)	0.30 (<0.001)
Calcium	0.24 (<0.001)	0.19 (0.002)	0.05 (0.570)
Phosphorus	0.06 (0.243)	0.07 (0.296)	0.01 (0.979)
Hemoglobin	0.23 (<0.001)	0.30 (<0.001)	0.06 (0.454)
hsCRP	−0.55 (<0.001)	−0.46 (<0.001)	−0.45 (<0.001)

AKI, acute kidney injury; APACHE II score, Acute Physiology and Chronic Health Evaluation II score; hsCRP, high sensitivity C-reactive protein.

Full model includes age, gender, diabetes, dialysis, RIFLE stage, APACHE II score, albumin, cholesterol, and hsCRP.

**Table 3:** Hazard ratios (HRs) and 95% confidence intervals (CIs) of 90-day mortality according to the four *a priori*-selected groups of serum prealbumin in all 340 patients with AKI.

	<10 (mg/dl)		10 to<15 (mg/dl)		15 to<20	≥20 (mg/dl)	
	HR (95% CI)	*P*	HR (95% CI)	*P*	(mg/dl)	HR (95% CI)	*P*
Unadjusted	4.10 (2.28, 7.38)	<0.001	3.13 (1.81, 5.44)	<0.001	1.0 (reference)	0.95 (0.47, 1.91)	0.886
Model 1	3.96 (2.20, 7.16)	<0.001	3.04 (1.74, 5.32)	<0.001	1.0 (reference)	0.92 (0.46, 1.87)	0.832
Model 2	3.67 (1.86, 7.24)	<0.001	2.51 (1.39, 4.53)	0.002	1.0 (reference)	0.91 (0.45, 1.85)	0.799
Model 3	2.55 (1.18, 5.49)	0.02	1.29 (0.57, 2.91)	0.55	1.0 (reference)	0.82 (0.38, 1.74)	0.607

AKI, acute kidney injury.

Variables of model 1 include age and gender.

Variables of model 2 include age, gender, hypertension, APACHE II score, and continuous renal replacement therapy.

Variables of model 3 include age, gender, hypertension, APACHE II score, continuous renal replacement therapy, serum albumin, white blood cell count, and serum high-sensitivity C-reactive protein.

**Table 4:** Hazard ratios (HRs) and 95% CIs of 90-day mortality according to the 3 *a priori*-selected groups of change in serum prealbumin in 315 patients and in 203 patients with serum prealbumin ≥15 mg/dL at the time of AKI diagnosis.

	Unadjusted		Model 1		Model 2		Model 3	
	HR (95% CI)	*P*	HR (95% CI)	*P*	HR (95% CI)	*P*	HR (95% CI)	*P*
Categories of change in prealbumin (mg/dl)								
<−4 (n = 72)	1.63 (1.00, 2.67)	0.051	1.70 (1.03, 2.77)	0.037	1.68 (1.02, 2.76)	0.040	1.79 (1.06, 3.03)	0.030
−4 to <4 (n = 167)	1.0 (reference)		1.0 (reference)		1.0 (reference)		1.0 (reference)	
≥4 (n = 76)	0.51 (0.26, 1.02)	0.058	0.59 (0.29, 1.18)	0.137	0.52 (0.26, 1.06)	0.072	0.54 (0.25, 1.15)	0.113
Categories of change in prealbumin ≥15 mg/dl								
<−4 (n = 62)	2.59 (1.26, 5.34)	0.010	3.16 (1.47, 6.81)	0.003	3.96 (1.60, 9.81)	0.003	3.85 (1.55, 9.52)	0.004
−4 to<4 (n = 110)	1.0 (reference)		1.0 (reference)		1.0 (reference)		1.0 (reference)	
≥4 (n = 31)	0.29 (0.04, 2.21)	0.232	0.41 (0.11, 1.46)		0.48 (0.12, 1.88)	0.293	0.46 (0.21, 1.82)	0.269

AKI, acute kidney injury; APACHE, Acute Physiology and Chronic Health Evaluation.

Variables of model 1 include age and gender.

Variables of model 2 include age, gender, hypertension, APACHE II score, and continuous renal replacement therapy.

Variables of model 3 include age, gender, hypertension, APACHE II score, continuous renal replacement therapy, serum albumin, white blood cell count, and serum high-sensitivity C-reactive protein.

The Cox proportional hazard model was used to calculate unadjusted and adjusted hazard ratio of 90-day mortality.

**Table 5:** The total integrated discrimination index (IDI) and net reclassification index (NRI) for adding prealbumin and its change to predict the 90-day survival.

Model	c Index (SE)	Absolute IDI (95% CI)	P	NRI (95% CI)	P
Conventional covariates	0.66 (0.04)				
Prealbumin	0.70 (0.04)	0.03 (−0.00, 0.11)	0.100	0.17 (−0.12, 0.36)	0.189
Prealbumin and its changes	0.73 (0.05)	0.08 (0.01, 0.18)	0.031	0.29 (0.03, 0.51)	0.040

Conventional covariates: serum creatinine, albumin, cholesterol and high sensitivity C-reactive protein.
